# 

**Published:** 1899-01-28

**Authors:** 


**Notices of Births, Deaths, and Marriages** are inserted in this column at a charge of 2s. 6d. for an announcement not exceeding four lines.

### NOTICE TO CORRESPONDENTS.

All MS., Letters, Books for Review, and other matters intended for the Editor should be addressed The Editor, "The Hospital," The Hospital Building, 28 & 29, Southampton Street, Strand, W.C.

The Editor cannot undertake to return rejected MS., even when accompanied by stamped directed envelope.

**Notice to Advertisers and Subscribers.**

All Advertisements, Orders for Copies of The Hospital, and Business Communications should be addressed to **THE MANAGER (Not to the Editor),** The Hospital, The Hospital Building, 28 & 29, Southampton Street, Strand, London, W.C.

The Cost of Subscription, post free, is as follows:—Per week, 2½d.; per quarter, 2s. 3d.; per half-year, 5s. 3d.; per annum, 10s. 6d. Foreign:—Per annum, 15s. 2d., payable, in all cases, in advance.

**NOTICE.—Vol. XXIV. of "The Hospital" (April, 1898, to September, 1898), in handsome cloth binding, is now on sale at the Office of this Journal, price 7s. 6d. Cases for binding the Numbers contained in this Volume 1s. 6d. Index to Vol. XXIV., 2½d., post free.**

**The Monthly Part for February will be ready on February 1st. Price 6d., by post 8d.**

### BOOKS RECEIVED.

THE SCIENTIFIC PRESS.

"Poison Romance and Mystery." By C. J. S. Thompson.

JOHN WRIGHT AND CO.

"Golden Rules of Surgical Practice." By E. Harry Fenwick, F.R.C.S.

"Golden Rules of Gynæcology." By S. Jervois Aarons, M.D.

"Golden Rules in Obstetric Practice." By W. E. Fothergill, M.A., B.Sc., M.D.

SWAN SONNENSCHEIN AND CO.

"Sanatoria for Consumptives in Various Parts of the World." By F. Rufenacht Walters, M.D., M.R.C.P. With an introduction by Sir Richard Douglas Powell, Bart., M.D., F.R.C.P.

"THE LOCAL GOVERNMENT JOURNAL" OFFICE.

"The Local Government Annual: An Official Directory, 1899." Edited by S. Edgecombe Rogers.

### The Student of Medicine.

### APPOINTMENTS.

H. C. Bennett, M.B., M.R.C.S., L.R.C.P., appointed House Physician to the Sunderland Infirmary. J. A. Bonnin, M.B., Ch.B.Adelaide, F.R.C.S.Eng., appointed House Physician to the East London Hospital for Children. J. A. Browne, L.R.C.S.I., appointed Assistant Ophthalmic Surgeon to the Belfast Royal Hospital. S. G. Champion, M.B., C.M., appointed Out-door Surgeon to the Leith Hospital. C. G. Cory, M.R.C.S., L.R.C.P.-Lond., appointed Medical Officer for the Seventh District of the Newmarket Union. L. A. Crooks, M.B., C.M., appointed House Surgeon to the Leith Hospital. A. H. P. Dawnay, M.R.C.S.Eng., L.R.C.P.Lond., appointed Anæsthetist to the Royal Orthopædic Hospital. C. Fenwick, M.R.C.S., L.R.C.P., L.S.A., appointed Surgeon to the South Metropolitan Gas Company. J. Fergus, L.R.C.P., L.R.C.S.Edin., appointed Medical Officer for the Appleton Wiske District of the Northallerton Union. A. G. R. Foulerton, F.R.C.S.Eng., D.P.H.Camb., F.C.S., appointed Bacteriologist to the Middlesex Hospital. J. Gaitskill-Churton, M.R.C.S., L.R.C.P., appointed Junior House Surgeon to the Stanley Hospital, Liverpool. A. Goodall, M.B., Ch.B., appointed Assistant House Surgeon to the Leith Hospital. F. R. Greenwood, M.R.C.S.Eng., L.R.C.P.Lond., appointed Resident Surgical Officer at Birmingham and Midland Free Hospital for Sick Children. E. S. Hamer, L.R.C.P., L.R.C.S.Edin., appointed Medical Officer for the Tyldesley District of the Leigh Union. C. O. Hawthorne, M.B.Glasg., appointed Medical Superintendent of the Medical Graduates' College and Polyclinic, Chenies Street. R. G. Henderson, M.A., M.B, Ch.B.Aberd., appointed Senior House Surgeon to the Stanley Hospital, Liverpool. W. J. Hirst, M.B.Lond., appointed House Surgeon to the Hospital and Dispensary, Newark-upon-Trent. J. B. Jamieson, M.B., C.M., appointed House Physician to the Leith Hospital. J. E. St. G. Johnstone, M.B., appointed Medical Officer for the Eldersfield District of the Upton-upon-Severn Union. S. J. J. Kirby, M.D.Brux., L.R.C.P., L.M.Edin., M.R.C.S.Eng., and L.S.A.Lond., appointed Medical Officer of Health to the Hoxne Rural District, and Medical Officer and Public Vaccinator of the Fressingfield District of the Hoxne Union. F. H. Moore, L.R.C.P., L.R.C.S.Irel., appointed Medical Officer for the Sibsley District of the Boston Union. J. E. Peirce, M.R.C.S.Eng., L.S.A., appointed Medical Officer and Public Vaccinator to the Bewley District of King's Norton Union. C. J. Perrott, L.R.C.P., L.R.C.S.Irel., appointed Medical Officer for the Oldland District of the Keynsham Union. R. D. Thomas, L.R.C.P., L.R.C.S., appointed Medical Officer for the Guilsfield District of the Llanfyllin Union. R. A. Young, M.D., B.Sc.Lond., M.R.C.P., appointed Medical Registrar to the Middlesex Hospital.

### "THE BURDETT SERIES" OF POPULAR TEXT-BOOKS ON NURSING.

Small crown 8vo., cloth. 1s. each.

No. 1.—**PRACTICAL HINTS ON DISTRICT NURSING.** By AMY HUGHES.

"The whole book bristles with good advice and common-sense. It should be in the hands of every district nurse."—*British Medical Journal.*

No. 2.—**THE MATRON'S COURSE.** An Introduction to Hospital and Private Nursing. By Miss S. E. ORME.

"Short practical lectures . . . readable and instructive . . . will prove useful to all who study them."—*Scotsman.*

No. 3.—**THE MIDWIVES' POCKET-BOOK.** By HONNOR MORTEN, Author of "How to Become a Nurse, and How to Succeed," &c., &c.

"The little book will admirably serve its purpose."—*Glasgow Herald.*

No. 4.—**FEVERS AND INFECTIOUS DISEASES:** their Nursing and Practical Management. By WILLIAM HARDING, M.D. Ed., M.R.C.P., Lond.

"A surprising amount of terse and perspicuous information. We heartily commend the book to nurses and to mothers of families."—*Aberdeen Free Press.*

No. 5.—**NOTES ON PHARMACY AND DISPENSING FOR NURSES.** By C. J. S. THOMPSON.

"The descriptions of the various processes are clear, the rules are to the point, and the pages are full of useful information."—*The Hospital.*

No. 6.—**THE RATIONAL USE OF ANTISEPTICS IN MIDWIFERY PRACTICE.** By JAMES MORRISON, M.D., M.B., M.R.C.S., L.R.C.P. (Lond.). [*In preparation.*

No. 7.—**CARE OF CONSUMPTIVES.** By W. H. DAW, M.R.C.S., L.R.C.P., Lond.

No. 8.—**NURSING OF SICK CHILDREN.** By J. D. E. MORTIMER, M.B., F.R.C.S., L.S.A. [*In preparation.*

No. 9.—**MENTAL NURSING.** By WILLIAM HARDING, M.D., Ed., M.R.C.P., Lond. [*In preparation.*

London: THE SCIENTIFIC PRESS, LTD.,
28 & 29, Southampton Street, Strand, W.C.

